# Parkinson’s Disease: Diagnostic Challenges Amidst Transdiagnostic and Overlapping Mental Health Symptoms

**DOI:** 10.7759/cureus.36661

**Published:** 2023-03-25

**Authors:** Jayakrishna S Madabushi, Mayank Gupta, Brett Pearce, Nihit Gupta

**Affiliations:** 1 Psychiatry, Alabama College of Osteopathic Medicine, Birmingham, USA; 2 Psychiatry and Behavioral Sciences, Southwood Psychiatric Hospital, Pittsburgh, USA; 3 Psychiatry, LSU New Orleans Psychiatry Training Program, New Orleans, USA; 4 Psychiatry, University of West Virginia, Glen Dale, USA

**Keywords:** anhedonia, alexithymia, mood disorder, depression, parkinson's disease

## Abstract

Parkinson’s disease (PD) is a complex neurodegenerative disorder with heterogeneous clinical presentations. Given the ambiguity of its overlapping symptomatology and concomitant atypical motor and neuropsychological symptoms its early diagnosis is clinically challenging. It is often missed since low mood, anhedonia, lack of motivation, and psychomotor retardation are commonly reported in individuals with PD. When alexithymia is the predominant symptom, the knowledge to discriminate between apathy, anhedonia, and alexithymia is critical to avoid misdiagnosis.

## Introduction

Parkinson’s disease (PD) is a complex progressive neurodegenerative condition that is uncommon in younger than 40, and the incidence of the disease increases rapidly over 60 years, with a mean age at diagnosis of 70.5 years [1]. The initial clinical presentation is highly varied with many reports of the presence of non-motor symptoms and the absence of distinctive symptoms leading to diagnostic challenges [2]. Although there is the availability of biomarkers in clinical settings, the diagnosis is clinically based on history and neurologic examinations without the routine use of gold standard tests [3]. The overlapping and trans-diagnostic nature of non-motor symptoms, higher psychiatric comorbidity, challenge differentiating between apathy, and anhedonia are major reasons for diagnostic uncertainty [4]. While symptoms of apathy and anhedonia could coexist, without an established diagnosis of PD, treating these symptoms in individuals with PD have poor clinical outcomes [5]. There are up to 50% of patients with PD have depressive symptoms, and often presents as an initial symptom [6]. Likewise, apathy is highly prevalent ranging from 17% to 62% in individuals with PD. Alexithymia is also frequently seen in PD but not widely reported in the empirical literature [7]. Many of these patients are referred for psychiatric consultation for the treatment of depressive symptoms. Therefore, it is imperative to understand the nuances of these clinical symptoms and be able to discriminate to make an accurate diagnosis. We, therefore, present a unique clinical case to illustrate complex and atypical presentations in real-world settings to underscore these challenges and strategies to improve diagnostics and overall outcomes. 

## Case presentation

A 63-year-old female was referred by PCP for treatment-resistant depression for psychiatric consultation. Per PCP’s reports, the patient presented with depressive symptoms, and she was tried on selective serotonin reuptake inhibitors (SSRSI) (Fluoxetine and Escitalopram), and the Serotonin, and norepinephrine reuptake inhibitors (SNRI) (Venlafaxine and Duloxetine) and Bupropion. The chronology of her psychopharmacological interventions is as follows; she was initially initiated on Fluoxetine 20 mg once daily for six weeks and then increased to 30 mg for four additional weeks with no response. She then switched to Escitalopram 10 mg once daily for three weeks and stopped due to lack of response. She also started on Duloxetine 60 mg for four weeks without any optimal response. She then was started on Venlafaxine XR 75 mg for three weeks which was titrated up to 225 mg which was maintained for eight weeks. Subsequently referred to the Psychiatric Clinic, where she reported having lost interest in everything and stopped doing most of her activities. She stopped going to her business. She also mentioned that she is not doing her makeup which was always interested in. She reported that her main concern was that her brother was getting irritable and frustrated with her for not showing interest in any activities. She reports no previous psychiatric illness. Her brother expressed his frustration that she is not showing interest in anything including daily chores. He reported that he was trying to encourage her, but she was not showing any interest. All these concerns were reported by the patient’s brother, while she did not report any specific symptoms therefore the review of psychiatric symptoms was negative.

During the initial appointment, she was presenting with symptoms of clinical depression with predominant anhedonia and poor response to the previous medication. At this point, her Venlafaxine extended release was titrated up to 300 mg and augmented with Aripiprazole 5 mg. The patient at six weeks for a follow-up reported no improvement in her symptoms and worsening of her functioning. The patient was compliant with medication. The patient reported cognitive slowing after starting this combination of medications. She also expressed her frustration that she could not understand why her brother and everyone were thinking that she was depressed, but in fact, she had been feeling like not have emotions at all. She reported that she was not feeling happy in situations when she usually feels happy, but rather she was feeling “flat.” Her brother also pointed out that she was not showing any emotion and she was not showing anger as well which he reported making all the family members feel frustrated.

The patient expressed her wish to stop anti-depressants as she did not find them effective. The clinical team tapered her medication before stopping and in the subsequent follow-up appointment, the patient reported that there is worsening in her “slowness.” On further inquiry, she noticed some slowness in her activity including walking for the last six months. She also reported that she is feeling more “flat” in her emotions after started taking a combination of Venlafaxine and Aripiprazole. The patient’s brother reported that her facial movements became slow as well as if she was wearing a mask. During the review, she was not showing any emotional reactivity and reported feeling annoyed about feeling flat.

An atypical case of PD was suspected; however, there were no reports of any tremors, rigidity, or any other symptoms. The neurologist consulted reported slowness in gait and her facial movements. Complete neurological work including heavy metal screening and neuroimaging studies were negative. After discussing with Neurology, the patient was initiated on Carbidopa/levodopa 25/100 three times daily after educating them about the atypical presentation of PD. The patient at four weeks follow-up came with full makeup on and was cheerful and happy. She reported have got all her ‘’ lost emotions ‘came back, and improvement in physical and mental slowness. At six months and she maintained improvement in her symptoms and functioning.

## Discussion

PD is a progressive nervous system disorder that affects movement and can also have non-motor symptoms. Depression is a common non-motor symptom of PD, and it is estimated that up to 50% experience depression at some point during their illness [8]. In PD, depression can present in different ways and at different stages of the disease. In the early stages of the disease, depression may be one of the first symptoms that appear, even before the motor symptoms become apparent. In the later stages of the disease, depression may be a result of the progressive loss of independence and mobility, as well as the impact of the disease on the individual's quality of life. Apathy and anhedonia are two related but distinct symptoms that can occur in a variety of mental health conditions. Apathy refers to a lack of motivation or interest in activities that were once enjoyed and is characterized by a lack of energy, initiative, and interest in social interactions. Apathetic individuals may seem indifferent or detached and may not initiate or participate in activities or conversations. Anhedonia, on the other hand, refers to the inability to experience pleasure or enjoyment. With anhedonia, individuals may still engage in activities, but they do not derive pleasure or satisfaction from them. Differentiating between apathy and anhedonia can be challenging, as they can occur together, and both symptoms can manifest as a lack of motivation and interest in activities. However, it's important to differentiate between them as they have different causes, implications, and treatment options. Standardized instruments like the Apathy Evaluation Scale (AES) and the Snaith-Hamilton Pleasure Scale (SHAPS) are often used to assess the severity of apathy and anhedonia [9]. 

Alexithymia, on the other hand, is characterized by difficulty in identifying and expressing one's emotions. While anhedonia can be a symptom of certain mental health conditions like depression, Alexithymia may be a personality trait, autism, schizophrenia, and even reported in PD [10]. It is very challenging for an individual to cope with Alexithymia as it causes various issues like difficulty identifying different types of feelings, difficulty recognizing facial cues, rigid imagination, constricted style of thinking, hypersensitive to physical sensations, and detached or tentative connection to others. In the context of this case report, the patient did not report specifically about anhedonia which is the inability to feel pleasure or lack of interest. It is her brother’s main concern that she was not showing any interest. Her main concern was that she was not having any emotions including sadness, anger, or happiness, and her flat affect was making her feel frustrated. This could be also confused with apathy, however phenomenologically it is considered an ego-syntonic and not an ego-dystonic symptom. What she described met the criterion for alexithymia as she was primarily reporting not experiencing any emotion; however, differentiating among these symptoms is often challenging.

There are a few other organic causes of depression in the geriatric population without a prior history of depression including certain neurological disorders, such as PD, Alzheimer's disease, and stroke. Chronic medical conditions, such as diabetes, heart disease, and cancer, medications, like opioids and sedatives, vitamin deficiencies, particularly in vitamin B12 and folate, and sleep disorders, such as insomnia and sleep apnea, may contribute to depressive illness.

There are ways to screen for PD with non-motor symptoms. A neurological examination is to assess for signs of PD, such as tremors, stiffness, and balance problems. If in doubt then, Unified Parkinson's Disease Rating Scale (UPDRS) and the Hoehn and Yahr scale, can be used to assess the severity of Parkinson's disease symptoms. Questionnaires, such as the Parkinson's Disease Sleep Scale (PDSS) and the Non-Motor Symptoms Scale (NMSS) can be used to assess the non-motor symptoms of Parkinson's disease [11,12]. 

Although typical depressive symptoms are common in PD, this case demonstrates that atypical symptoms of depression may be seen in PD. In terms of descriptive phenomenology Apathy is defined as a lack of feeling or emotion and is often considered ego-syntonic. However, the symptoms of depressive illness are mostly ego-dystonic. Anhedonia which is a lack of pleasure is one of the typical symptoms of depression. Bradyphrenia, slowness in thinking and processing of information is also well reported in PD [13], Similarly, emotional akinesia, a reduced capacity to experience emotions or an inability of emotional experiences to influence intentional and attentional systems, was reported in individuals with PD [14]. This may be like Alexithymia which is difficulty in identifying and expressing emotions [15]. It also constitutes a struggle to communicate their emotions to others. It may cause a lot of distress to the individual and family members as well. Our patient presented with the symptoms of alexithymia and responded well with Carbidopa/Levodopa. Ten studies reported prevalence of Alexithymia is double in PD when compared to the general population [16]. Table 1 provides key differences in the symptoms present in these conditions.

**Table 1:** Summary of differences between apathy, anhedonia, and alexithymia A transdiagnostic approach is needed to develop contextual understanding of these common syndromes [17] and remains critical to improving outcomes in serious neuropsychiatric disorders [18,19].

Apathy	Anhedonia	Alexithymia
Apathy is a lack of feeling, emotion, interest, or concern about something.	Anhedonia is the inability to experience pleasure from activities that are normally found enjoyable.	Alexithymia is a condition characterized by difficulty identifying and expressing emotions.
dementia, Parkinson's disease, stroke, brain injury, depression, and schizophrenia.	Major depressive disorder, schizophrenia, can also be seen in individuals with substance abuse disorders and certain medical conditions such as HIV/AIDS.	Autism, depression, anxiety disorders, post-traumatic stress disorder (PTSD), Parkinson’s disease and somatoform disorders.

## Conclusions

PD is a progressive nervous system disorder with broad symptomatology encompassing both motor and non-motor symptoms. While the symptoms of alexithymia and apathy may coexist in patients with PD prior to an established diagnosis, therefore symptomatic treatment results in poor clinical outcomes. While depressive symptoms are common in PD, they often overlap with other symptoms. Neuro-vegetative symptoms like psychomotor retardation, blunted affect, and anhedonia which are more commonly associated with affective illness can closely mimic the alexithymia, bradykinesia, hypomimia, and apathy associated with PD. The atypical presentation of PD is not uncommon, however, when alexithymia is predominant at the initial presentation, distinguishing non-motor symptoms of PD from primary psychiatric disorders remains a challenge.
